# Treatment of Misophonia with Cognitive Behaviour Therapy: A Case Report

**DOI:** 10.1192/j.eurpsy.2024.880

**Published:** 2024-08-27

**Authors:** K. B. Avanoğlu, C. Kılıç

**Affiliations:** ^1^Department of Psychiatry, Yalova State Hospital, Yalova; ^2^Department of Psychiatry, Hacettepe University Hospitals, Ankara, Türkiye

## Abstract

**Introduction:**

Misophonia is a condition characterized by extreme emotional reactions, such as irritation or anger, triggered by specific sounds. Despite its prevalence, there is a lack of evidence-based treatment methods for misophonia.

**Objectives:**

This case report aims to explore the effectiveness of combining psychoeducation with Cognitive Behavioral Therapy (CBT) in the treatment of a misophonic patient. The focus is on reducing the patient’s emotional distress and improving their quality of life.

**Methods:**

The patient is a 28-year-old woman employed as a salesperson in a busy city. Mouth smacking, gum chewing and clock ticking are the sounds that bother her the most. She has never used any medications or attempted any methods to alleviate her misophonia. Neither she, nor her family has a history of a psychiatric disorder.

The therapeutic intervention spanned eight sessions, each lasting around half an hour. The first two sessions, a patient history was taken and Mısophonia Interview Scale (MIS) was conducted. MIS comprised the Misophonia Checklist (MCL), which involved reading fifty misophonic sounds to the patient one by one. She then rated her discomfort in response to each sound on a four-point Likert-type scale. From the MCL responses, a total severity score (Misophonia Total Score - MTS), was calculated.

The treatment commenced with a psychoeducational component focused on enhancing the patient’s comprehension of misophonia. This phase aimed to elucidate the neurobiological underpinnings of the condition, common triggers, and the emotional reactions associated with it.

Then, CBT was employed to identify and challenge the patient’s negative automatic thoughts (NATs) linked to her misophonia. Three sessions primarily concentrated on identifying and managing NATs associated with her misophonia. These sessions equipped the patient with the skills to recognize and confront NATs through structured discussions and practical assignments.

The last three sessions centered on exposure therapy, with the goal of reducing emotional and physiological responses to triggers. Homework assignments during this phase encouraged the patient to independently practice exposure exercises.

**Results:**

The initial MTS was 54, indicating significant distress. After the interventions, the final MTS decreased to 35 and the impact of misophonic symptoms on her life decreased from severe to moderate.

**Conclusions:**

Misophonia is a challenging disorder to treat due to its limited evidence-based interventions. This case report demonstrates that a combination of psychoeducation and CBT methods may hold promise in managing misophonic symptoms. However, it is essential to acknowledge the need for further research in this area, as misophonia’s treatment strategies require more robust empirical support. This case highlights the potential benefits of psychoeducation and CBT, emphasizing the need to explore and develop effective treatments for this debilitating condition.

**Disclosure of Interest:**

None Declared

